# A systematic review of community based hepatitis C treatment

**DOI:** 10.1186/s12879-016-1548-5

**Published:** 2016-05-16

**Authors:** Amanda J. Wade, Vanessa Veronese, Margaret E. Hellard, Joseph S. Doyle

**Affiliations:** Centre for Population Health, Burnet Institute, Melbourne, Australia; School of Public Health and Preventive Medicine, Monash University, Melbourne, Australia; Department of Infectious Diseases, The Alfred Hospital, Melbourne, Australia; Department of Medicine, University of Melbourne, Melbourne, Australia

**Keywords:** Hepatitis C, Community-based, Opioid substitution, Treatment, Models of care

## Abstract

**Background:**

Hepatitis C virus (HCV) treatment uptake globally is low. A barrier to treatment is the necessity to attend specialists, usually in a tertiary hospital. We investigate the literature to assess the effect of providing HCV treatment in the community on treatment uptake and cure.

**Methods:**

Three databases were searched for studies that contained a comparison between HCV treatment uptake or sustained virologic response (SVR) in a community site and a tertiary site. Treatment was with standard interferon with or without ribavirin, or pegylated interferon and ribavirin. A narrative synthesis was conducted.

**Results:**

Thirteen studies fulfilled the inclusion criteria. Six studies measured treatment uptake; three demonstrated an increase in uptake at the community site, two demonstrated similar rates between sites and one demonstrated decreased uptake at the community site. Nine studies measured SVR; four demonstrated higher SVR rates in the community, four demonstrated similar SVR rates, and one demonstrated inferior SVR rates in the community compared to the tertiary site.

**Conclusion:**

The data available supports the efficacy of HCV treatment in the community, and the potential for community based treatment to increase treatment uptake. Whilst further studies are required, these findings highlight the potential benefit of providing community based HCV care – benefits that should be realised as interferon-free therapy become available.

(PROSPERO registration number CRD42015025505).

**Electronic supplementary material:**

The online version of this article (doi:10.1186/s12879-016-1548-5) contains supplementary material, which is available to authorized users.

## Background

Each year in Australia less than 2 % of people infected with hepatitis C virus (HCV) are treated and globally treatment uptake rates are similarly low [1]. Barriers to HCV treatment include; difficulty in accessing a treatment service, not being offered treatment once in a treatment service and toxic pegylated interferon based treatment with poor efficacy [2–5]. Stigma is also a significant barrier to treatment in health care settings [6].

Fortunately the HCV treatment landscape is changing; pegylated interferon, ribavirin and protease inhibitor regimens of 6–12 months duration, which generate serious adverse effects in about 10 % of people and achieve cure in only 70 % are being replaced by all oral, well tolerated interferon free, direct acting antiviral (DAA) therapy, often for 12 weeks duration, with cure in more than 95 % [7, 8]. Although treatment tolerability and efficacy as a barrier to HCV treatment has been overcome, in the vast majority of countries HCV antiviral costs remain prohibitive. For DAA therapy to have maximum impact on the HCV epidemic, it must be affordable and accessible. To date, in most developed and many developing countries specialist physicians have provided HCV treatment, usually from tertiary hospital outpatient clinics. Such clinics often have rigid appointment scheduling and do not always provide multidisciplinary care. The reassuring safety profile and high efficacy of DAA therapy means HCV treatment could now be provided in a diverse range of clinical settings. HCV treatment could be provided in community-based clinics, including opioid substitution therapy (OST) clinics or using telehealth, with a variety of service providers including nurses, general practitioners and specialists. Increasing treatment accessibility may significantly improve HCV treatment uptake and cure, but a key issue is a lack of quality information about which model of care is most efficacious.

The Australian government has recently made a landmark decision to fund DAA therapy for every Australian infected with hepatitis C from 1st March 2016 [9]. In addition, a new model of care will be implemented in order to facilitate access to treatment. General practitioners will be able to prescribe DAA, albeit after authorization from a specialist [10]. As the new Australian model of care unfolds, it is timely to reflect upon the available evidence regarding hepatitis C treatment in the community.

To gain data that may inform HCV service delivery policy, we reviewed the literature to compare treatment uptake rates in community based treatment services with conventional tertiary services, and to compare sustained virological response (SVR) outcomes in patients treated with standard interferon with or without ribavirin, or pegylated interferon and ribavirin, in the community with patients treated in conventional tertiary settings.

## Methods

Published research was scanned by formal searches of three electronic databases (Medline, EMBASE and CINAHL) from January 2000 to July 2015. Search terms included “hepatitis C”, “antiviral agents”, “patient care management” and “healthcare delivery”. The full search strategy is detailed in the Additional file 1. Citations were screened and evaluated using the established inclusion and exclusion criteria at the abstract level by two operators (AW and VV), and relevant studies were retrieved as full manuscripts. Articles were restricted to English language.

1.1. Eligibility criteria

Inclusion criteria were:

(i) people with chronic HCV infection and;
(ii) provision of treatment for hepatitis C in the community and;
(iii) comparison with tertiary based services and;
(iv) measuring and reporting either treatment uptake or SVR outcomes.

Treatment could include pegylated interferon and ribavirin, with or without DAA or interferon-free. Health care provider could be a specialist or general practitioner or nurse; the use of telehealth was permitted.

Exclusion criteria were defined as:

(i) treatment of custodial populations or;
(ii) treatment of HIV-HCV co-infected populations or;
(iii) treatment of children or;
(iv) treatment in residential facilities (i.e. inpatient rehabilitation) or;
(v) modeling studies or;
(vi) papers assessing patient or practitioner knowledge or attitudes or;
(vii) papers published before 2000 because interferon ribavirin combination therapy was only licensed in 1998 and antiviral treatment was exclusively delivered in tertiary care.

1.2. Definitions and end-points
  A community service was defined as a medical service that was not a tertiary hospital or academic facility, including primary care clinics that may provide opiate substitution therapy (OST) and private practice. Treatment uptake was defined as proportion of HCV infected patients at service that received a prescription for HCV treatment. Cure was defined as sustained virologic response (SVR) at week 12 or 24 post cessation or completion of HCV treatment.

1.3. Study selection
  Using inclusion and exclusion criteria, identified abstracts were assessed for relevance by two researchers (AW and VV). Variations in citation assessment were resolved by a third reviewer (JD). Full text papers were then retrieved for review. If further data were required to classify a full text paper the authors were contacted. The following information was obtained for each article; authors, year of publication, country of origin, number of subjects, healthcare delivery structure, treatment uptake rate, SVR rate. For studies that measured SVR rate the following additional data was extracted: proportion with genotype 1 infection, proportion with HIV co-infection, prior treatment history and proportion with advanced fibrosis.

A narrative review of the included studies was performed. This review is registered with the PROSPERO database (registration number CRD42015025505).

## Results

**Fig. 1:**
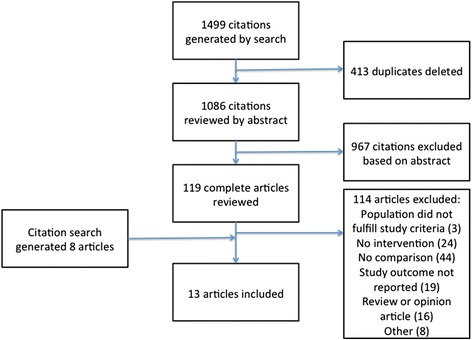
Flow diagram of study analysis

The flow diagram of the study analysis is shown in Fig. 1. The search generated 1499 citations, 413 duplicates were then deleted. Of the remaining 1086 citations, 967 were excluded based on the abstract. Full text articles were retrieved for 119 citations. A further 8 articles were included after citations searching. Thirteen of the 127 articles fulfilled the inclusion criteria. A summary of data from included articles is shown in Table 1, below.

**Table 1:** Summary of included studies

Study	Year	Country	Design	Intervention	Facility	n	Rx uptake	SVR
							n (%)	n (%)
Arora	2011	USA	Prospective cohort study of treatment outcome	Telehealth to support primary care (in community and prison)	Tertiary	146		84/146 (58)
					Primary total (Prisoners)	261 (106)		152/261 (58)
Bruce	2012	USA	Randomised controlled trial of treatment uptake and outcome	Directly observed therapy in OST clinic vs self administered treatment in tertiary clinic	Tertiary	9	4/9 (44)	1/4 (33)
					Primary	12	12/12 (100)	6/12 (75)
Chen	2014	Taiwan	Prospective cohort study of treatment outcome	Telecare	Tertiary	150		99/150 (66)
					Telecare	148		102/148 (69)
Gigi	2013	Greece	Retrospective cohort study of treatment uptake	Rx in OST clinic	Tertiary	643 Ab+	276/643 (43)	
					Primary	204 Ab+	17/204 (8)	
Jou	2013	USA	Retrospective cohort study of treatment outcome	Analysis of data by Rx site	Academic	1905		760/1905 (40)
					Community	1165		455/1165 (39)
Kramer	2010	USA	Retrospective cohort study of treatment uptake		Specialist clinic	24,853	3537 (14)	
					Primary Care clinic	1929	251 (13)	
Kuo	2015	Taiwan	Prospective cohort study of treatment uptake and outcome	Rx in community	Pre intervention	18	4/18 (22)	2/4 (50)
					Post intervention		3/16 (19)	3/3 (100)
Moriarty	2001	New Zealand	Observational study of treatment uptake	Rx co-located at NSP site	Tertiary	51	1 (2)	
					Primary		4 (8)	
Moussalli	2010	France	Observational study of treatment uptake	Rx in OST clinic	Pre intervention	337	2/337 (0.6)	
					Post intervention		85/335 (25)	37/85 (44)
Myers	2011	Canada	Observational study of treatment outcomes		Academic	133		79/133 (59)
					Community	250		120/250 (48)
Nazareth	2013	Australia	Retrospective cohort study of treatment outcomes	Telehealth	Tertiary	528		311/528 (59)
					Telehealth	50		36/50 (72)
Niederau	2014	Germany	Prospective cohort study of treatment outcome	Analysis of adherence to guidelines by Rx site	Hospital based	621		290/621 (47)
					Private practice	3778		1744/3778 (46)
Rossaro	2013	USA	Retrospective cohort study of treatment outcomes	Telehealth	Tertiary	40		16/37 (43)
					Telehealth	40		21/38 (55)

*Rx* treatment, *OST* opioid substitution therapy, *Ab +* HCV antibody positive

Five of the included studies were from the US, two were from Taiwan and there was one study each from Australia, New Zealand, Greece, France, Germany and Canada. The interventions to provide treatment in the community were diverse and included telehealth and treatment provision from primary care clinics, opioid substitution therapy (OST) clinics or needle exchange programs. Treatment consisted of pegylated interferon and ribavirin in all studies but for Moriarty [11] and Gigi [12], in which standard interferon with or without ribavirin was also included.

### Studies which measured treatment uptake only (see Table 2)

**Table 2:** Summary of studies which investigated treatment uptake

Study	Study population and policy for initiating treatment (if included in publication)	Facility	N	OST (%)	Active illicit substance use	Treatment uptake
						n (%)
Bruce	HCV infection +/− HIV Attendance at OST clinic Rx according to published guidelines and the same in both facilities	Tertiary	9	100	Opioid negative on urine toxicology in past 30 days	4 (44)
		Primary	12	100	Opioid negative on urine toxicology in past 30 days	12 (100)
Gigi	HCV antibody positive Attended Liver clinic or OST clinic Policy for Rx initiation not published	Tertiary	643	0	Nil	276 (43)
		Primary	204	100	Nil	17 (8)
Kramer	HCV infection Designated Primary Care Provider Majority of care from one Veterans Affairs facility Rx indicated if more than portal fibrosis and no contraindications (including no active illicit drug use)	Specialist clinic	24,853	N/P	N/P	3537 (14)
		Primary clinic	1929	N/P	N/P	251 (13)
Kuo	HCV antibody positive Participation in screening program Pre-intervention Rx if: ALT >40 (once) and > F1 or HCV RNA positive Post intervention Rx if ALT >80 (twice) and > F1	Pre intervention	18	N/P	N/P	4 (22)
		Post intervention		N/P	N/P	3 (19)
Moriarty	HCV infection Attendance at outreach clinic Policy for Rx initiation not published	Tertiary	51	N/P	N/P	1 (2)
		Primary		N/P	N/P	4 (8)
Moussalli	HCV infection Attendance at OST Primary healthcare facility Rx if > F2 fibrosis	Pre-intervention	337	N/P	N/P	2 (0.6)
		Post intervention		N/P	N/P	85 (25)

*OST* opioid substitution therapy, *N/P* not provided, *Rx* treatment

Two studies investigated the outcome of treatment provision in opioid substitution clinics, and had different results. Moussalli et al. noted an increase in treatment uptake when provided at an OST clinic. Before treatment was available in the OST clinic two of 337 patients had commenced treatment for HCV. After treatment was made available in the OST clinic 85 patients commenced therapy, and of those patients 37 (44 %) achieved an SVR [13]. However, in a retrospective cohort study in Greece, only 17 of 204 HCV antibody positive patients (8 %) commenced treatment in an OST setting, compared to 276 of 643 patients (43 %) in a tertiary liver unit [12]. Of note, few HCV antibody positive patients in the OST clinic had HCV RNA testing performed - 33 of 204, of which 28 were positive. In comparison, 498 of the 643 HCV antibody positive patients in the tertiary liver unit were known to be HCV RNA positive.

A retrospective study of treatment uptake in a needle and syringe exchange program (NSEP) centre in New Zealand found of 51 HCV infected patients, four commenced treatment at the needle exchange centre, whilst only one patient commenced treatment at the hospital [11].

A large retrospective study in the US demonstrated that treatment uptake in primary care clinics 251 of 1929 patients (13 %) was similar to treatment uptake in specialist clinics 3537 of 24,853 (14 %) [14].

### Studies which measured treatment outcome only (see Table 3)

**Table 3:** Summary of trials which investigated treatment outcome

Study	Facility	N	Age (years)	Gender (% male)	Genotype 1 (%)	HIV infected n (%)	Prior treatment	Fibrosis assessment	Fibrosis result	SVR
									Mean or %	n (%)
Arora	Tertiary	146	45	45	57	0	Naive	APRI	0.938	84 (58)
	Telehealth	261	42	73	56	0			0.935	152 (58)
Bruce	Tertiary	9	43	67	67 (G1&4)	3 (33)	N/P	Biopsy (G1 only)	F4 33 %	1 (33)
	Primary	12	40	42	67 (G1&4)	3 (25)			F4 25 %	6 (75)
Chen	Tertiary	150	52	N/P	58	0	Naive		N/P	99 (66)
	Primary	148	47	N/P	61	0			N/P	102 (69)
Jou	Academic	1905	48	59	100	0	Naive	Biopsy	F3/4 10 %	760 (40)
	Community	1165	47	61	100	0			F3/4 11 %	455 (39)
Kuo	Preintervention	18	57	33	N/P	N/P	N/P		N/P	2 (50)
	Post intervention									3 (100)
Myers	Academic	133	46	70	49	0	Naïve & experienced	Biopsy	F4 14 %	79 (59)
	Community	250	46	64	55	0			F4 10 %	120 (48)
Nazareth	Tertiary	528	43	65	N/P	N/P	Naïve & experienced	Biopsy or Hepascore	F4 19 %	311 (59)
	Telehealth	50	46	50	60	N/P		Hepascore	F4 20 %	36 (72)
Niedaerau	Hospital based	621	N/P	N/P	100	N/P	N/P		N/P	290 (47)
	Private practice	3778	N/P	N/P	100	N/P			N/P	1744 (46)
Rossaro	Tertiary	40	54	55	65	0	Naive	Biopsy	F4 45 %	16 (43)
	Telehealth	40	51	48	65	0			F4 28 %	21 (55)

*N/P* not provided

Three cohort studies compared SVR rates obtained by standard care in a tertiary hospital with SVR rates obtained using telehealth (video-conferencing) to populations with poor access to specialist care i.e. in rural or remote areas, or prison. A large prospective study in the US demonstrated no difference in SVR between patients treated in tertiary care and patients treated by their primary care clinician with telehealth support (58 % in both groups) [15]. A smaller retrospective study in the US demonstrated similar results, with 43 % of tertiary patients obtaining an SVR compared to 55 % patients treated via telehealth [16]. A retrospective Australian study found 72 % of telehealth treated patients had an SVR compared to 59 % of tertiary treated patients [17]. All three studies demonstrate SVR rates achieved in telehealth care were similar or higher when compared to SVR rates achieved in tertiary care.

Chen et al., performed a study in which patients selected treatment delivered via telephone consultations provided from a health communication center, or treatment delivered conventionally in a hospital outpatient clinic, and detected no difference in SVR outcomes [18].

Three observational studies examined SVR outcomes of community based treatment. Jou retrospectively analysed results from a randomized control drug trial according to treatment site. SVR outcome were the same in the academic (40 %) and the community (39 %) sites [19]. Niederau also found similar SVR outcomes between treatment provided in a hospital with 290 of 621 patients (47 %) attaining SVR, and 1744 of 3778 patients (46 %) attaining SVR in private practice [20]. However, in an observational study in Canada lower rates of SVR were seen in community settings 120 of 250 patients (48 %), when compared to academic centres, 79 of 133 patients (59 %) [21]. Further analysis demonstrated the difference was due to lower SVR rates in patients infected with genotype 1 treated in the community.

### Studies which measured treatment uptake and outcome (see Tables 2 and 3)

Bruce et al. conducted a randomized clinical trial in which subjects on methadone in an OST clinic were randomized to receive modified directly observed treatment at the OST clinic or standard of care therapy at a tertiary liver clinic. Subjects treated at the OST clinic had directly observed therapy (DOT) for methadone, pegylated interferon and morning ribavirin doses, but self administered evening ribavirin. All 12 patients randomized to the OST clinic started treatment and six of eight patients (75 %) eligible to be assessed for SVR achieved SVR. In comparison four of the nine patients (44 %) randomized to standard of care commenced treatment and one of three patients (33 %) eligible to be assessed for SVR achieved an SVR [22].

A small Taiwanese study showed similar treatment uptake rates with tertiary care, four of eighteen patients (22 %) compared to three of 16 patients (19 %) commencing treatment when it was made available in the community [23]. SVR was achieved in two of four patients (50 %) in the tertiary facility and three of three patients (100 %) in the community facility.

## Discussion

This systematic review has identified publications, which contain a comparison between HCV treatment uptake rates or SVR outcomes in community and tertiary treatment services. Of the thirteen publications included, only one was a randomized controlled trial and the remainder were observational studies. The interventions that resulted in HCV treatment provision in the community were diverse, and included; telehealth, integrated HCV services in OST clinics or NSEP services, private medical practice and outreach services staffed by specialists or nurses.

Of the six studies that measured treatment uptake as an outcome (see Table 2), three demonstrated an increase in uptake at the community site [11, 13, 22]. Interestingly, two of these studies were conducted in OST clinics, and the third in a NSEP service. Two studies demonstrated similar treatment uptake rates between the community and tertiary services [14, 23]. The large study by Kramer et al. investigated the treatment uptake within the Veterans Affairs Healthcare in the United States according to whether treatment was provided from a primary care provider clinic or a specialist clinic. It is not known what proportion of the primary care provider clinics may have been OST providers as well. One study demonstrated decreased treatment uptake at the community site [12]. The authors attributed this difference to a difficulty in collaboration between OST staff and hospital based specialists.

The factors contributing to increased treatment uptake in the community sites varied according to the study; provision of non invasive fibrosis assessment (Fibrotest-Actitest) (Mousalli), multidisciplinary services (Mousalli, Moriarty, Bruce), modified directly observed therapy (Bruce) and gaining trust (Moriarty), led to improved management of HCV in the community setting.

Of the nine studies that measured SVR as an outcome (see Table 3), four demonstrated higher SVR rates in the community group [16, 17, 22, 23]. Possible reasons for this include that the community services were more convenient for the patients and offered a “one stop shop” where multiple needs could be met, or that HCV treatment was integrated into a developed patient-provider relationship. Another explanation is that the availability of multidisciplinary services may have helped mitigate factors associated with poor adherence or SVR outcomes in PWID such as unstable housing, poor social functioning and ongoing drug use [24]. Four studies demonstrated similar outcomes between the two treatment settings [15, 18–20]. One study from Canada in which 250 patients were treated in the community and 133 in an academic centre, demonstrated lower SVR rates in patients treated in the community [21]. The difference was due to SVR outcomes in genotype one patients only. Patient characteristics including level of fibrosis, rates of dose modification and treatment cessation for genotype one infected patients were similar in the community and academic sites, and an explanation for the difference in SVR between treatment sites was not apparent.

Modelling studies indicate that treatment uptake is the major limiting factor to substantial reductions in disease burden. Current treatment rates in Australia of 3 per 1000 PWID annually would need to be scaled up to 40 per 1000 PWID annually to halve HCV prevalence by 2030. [25]. The advent of DAA therapy has made the elimination of HCV a tangible concept since treatment is simple and well tolerated, but for this to be achieved a significant change in service delivery would be required, and has been undertaken.

Nine of ten studies reporting SVR outcomes demonstrated similar or superior SVR rates were achieved in the community. Further, findings in this review suggest that decentralising HCV services and providing HCV treatment in the community, particularly OST clinics, may increase treatment uptake. Numerous cohort studies conducted in OST clinics indicate that HCV treatment in this setting can be successful, even in the peginterferon based treatment era [19, 26, 27]. The key components of successful HCV treatment delivery in the community need to be identified, to inform policy and ensure that integrated services are adequately resourced.

This review was limited by the lack of published data that compares outcomes of HCV treatment delivered in the community with treatment delivered in conventional tertiary settings. Some studies included in the review have a small number of participants and therefore lack statistical power. There was only one small randomised controlled trial comparing community and tertiary based treatment, and this study also provided DOT to the patients in the community arm, rendering the relative contribution of both interventions difficult to assess. This review investigated interferon based HCV treatment and therefore the findings may not be applicable to HCV treatment with DAA. A large randomised controlled trial addressing the effect of community provision of HCV DAA treatment – the Prime Study based in Melbourne, Australia – is underway (clinicaltrials.gov NCT02555475). It is likely that any treatment outcome difference between hospital and community care may become less pronounced as treatment becomes easier with DAA therapy.

## Conclusion

In conclusion, this review demonstrates that the limited data available supports the safety of peginterferon based HCV treatment in the community, and the potential for community based treatment to increase treatment uptake. The paucity of high quality data available to assess the effect of HCV treatment in the community on HCV treatment uptake is striking. This variable is a key component in the hepatitis C cascade of care, and further studies are warranted to clarify how best to structure HCV service delivery in the era of DAA.

## Ethics approval and consent

Not applicable.

## Consent for publication

Not applicable.

## Availability of data and materials

The search strategy used to generate data, which supports the conclusions of this article, is included as an Additional file 1.

## Additional file

Additional file 1: Search Strategy for systematic review. (DOCX 85 kb)

## Abbreviations

DAA: direct acting antivirals
DOT: directly observed therapy
HCV: hepatitis C virus
NSEP: needle and syringe exchange program
OST: opioid substitution therapy
PWID: people who inject drugs
SVR: sustained virologic response
