# Supplementary material for: A systematic review of community based hepatitis C treatment
Source: BMC Infect Dis. 2016 May 16;16:202. doi: 10.1186/s12879-016-1548-5 (PMC4867528; doi:10.1186/s12879-016-1548-5)
Supplement: Additional file 1: — Search Strategy for systematic review. (DOCX 85 kb) [file 12879_2016_1548_MOESM1_ESM.docx]

**Search Strategy for systematic review**

**Database 1**

**Medline**

**Search1**

*Concept A1 Hepatitis C*

MeSH

- exp hepatitis C
- Hepacivirus

.tw.

- hepatitis C
- HCV

AND

*Concept A2 drugs used to treat HCV*

MeSH

- Antiviral agents/tu
- Ribavirin/tu
- Interferon-alpha/tu
- Interferons/tu

.tw.

- ribavirin
- (peginterferon or pegylated-interferon or peg-interferon or peyglated interferon)

AND

*Concept B community*

MeSH

- Patients care management
  - Comprehensive healthcare, nursing process, nursing assessment, nursing diagnosis, nursing research, nursing education research, nursing evaluation research, nursing methodology research, patient care planning, case management, critical pathways, patient centered care, primary health care, continuity of patient care, delivery of healthcare, delivery of healthcare- integrated, healthcare reform, health services accessibility, healthcare disparities, telemedicine, remote consultation, diseases management, medication therapy management, nurses practice patterns, patient care team, nursing team, patient navigation, physicians practice patterns, pint of care systems
- Exp nurses
- Community health services, community health nursing, community networks, community pharmacy services, preventive health services
- Physicians, general practitioners, hospitalists, physicians family, physicians primary care
- Opiate substitution treatment or substance-related disorders/dt,th or substance abuse, intravenous/dt,th

**Search 2**

*Concept C Hepatitis C drug therapy*

MeSH

- Hepatitis C/dt, th

AND

Concept D treatment outcome

MeSH

- Treatment outcome

AND concept B

**Search 3**

*Concept A1 and A2*

.tw.

- ((HCV or hepatitis C or hepacivirus) adj5 (treat* or therap* or manage*))

AND

*Concept B*

.tw.

- (communit* or nurse-led or general practi* or primary care or family practi* or family physician* )
- OR
- (integrate* adj3 (care or service))

**Search 1 + search 2 + search 3**

Limits

- Remove case reports, letters, comments, editorials, reviews, news, news articles
- English only

= 516 articles

**Database 2**

**Embase**

**Search 1**

*Concept A1 hepatitis C (treatment)*

Emtree

- hepatitis c/dt, th
- hepatitis c virus/dm

AND

*Concept A2 drugs used for HCV treatment*

Emtree

- ribavirin
- peginterferon
- interferon
- peginterferon alpha 2b
- peginterferon alpha 2a
- peginterferonalpha 2b plus ribavirin
- peginterferonalpha 2a plus ribavirin
- recombinant alpha 2a interferon
- recombinant alpha 2b interferon
- sofosbuvir
- lepidasvir
- paritaprevir
- ombitasvir
- dasabuvir
- daclastasvir
- boceprevir
- telaprevir
- simeprevir
- aliosporivir
- grazoprevir

AND

*Concept B community based service*

Emtree

- community care
- healthcare delivery
- nurse / clinical nurse specialist / nurse consultant / nurse practitioner
- nursing staff
- primary medical care
- healthcare access
- medical service
- health center
- community assessment
- patient care
- healthcare utilization
- health program
- telemedicine
- opiate substitution treatment / or substance abuse/dt, th or addiction/dt,th

**Search 2**

*Concept C hepatitis C*

Emtree

- exp hepatitis C
- exp hepatitis C virus

AND

*Concept D antiviral treatment*

Emtree

- antivirus agent / dt

AND

*Concept B community based service*

**Search 3**

*Concept A1 and A2*

.tw.

- ((HCV or hepatitis C or hepacivirus) adj5 (treat* or therap* or manage*))

AND

*Concept B*

.tw.

- (communit* or nurse-led or general practi* or primary care or family physician* or family practi*)
- OR
- (integrate* adj3 (care or service))

**Search 1 and search 2 and search 3**

Limits

- Remove conference abstract, editorial, letter, review,
- English only

= 633 articles

**Database 3**

**CINAHL**

**Search 1**

*Concept A1 hepatitis C and concept B1 treatment*

Cinahl terms

- exp hepatitis C drug therapy or therapy
- exp hepatitis C chronic drug therapy or therapy

AND

*Concept C community*

Cinahl terms

- health services research
- health services accessibility
- multidisciplinary care team
- nursing role or nurses or clinical nurse specialist or nurse practitioners or adult nurse practitioners
- primary healthcare – administration
- health care delivery
- healthcare delivery – administration
- primary healthcare
- integrative medicine
- community health centers or community health services or community health nursing
- substance abuse intravenous – drug therapy
- substance use disorders – drug therapy
- telemedicine

**Search 2**

*Concept A2 hepatitis C*

Cinahl terms

- exp hepatitis C
- hepatitis C chronic

AND

*Concept B2 treatment*

Cinahl terms

- exp antiviral agents therapeutic use
- ribavirin therapeutic use

AND

*Concept C community*

**Search 3**

*Concept A3 hepatitis C nursing*

Cinahl terms

- hepatitis C+/NU

**Search 4**

*Concept A1 and B1*

Title (TI) OR abstract (AB)

- ((HCV or hepatitis C or hepacivirus) N5 (treat* or therap* or manage*))

AND

*Concept C*

Title and abstract

- (communit* or nurse-led or general practi* or primary care or family practi* or family physician* )
- OR
- (integrate* N3 (care or service))

**Limits**

- English language
- Published between 2000 and current

= 350 articles
